# Supplementary material for: Human coronavirus OC43-elicited CD4+ T cells protect against SARS-CoV-2 in HLA transgenic mice
Source: Nat Commun. 2024 Jan 26;15:787. doi: 10.1038/s41467-024-45043-2 (PMC10817949; doi:10.1038/s41467-024-45043-2)
Supplement: Supplementary file 1 — Supplementary Information [file 41467_2024_45043_MOESM1_ESM.pdf]

**Human coronavirus OC43-elicited CD4$^+$ T cells protect against SARS-CoV-2 in HLA transgenic mice**

Rúbens Prince dos Santos Alves[1], Julia Timis[1], Robyn Miller[1], Kristen Valentine[1], Paolla Beatriz Almeida Pinto[1], Andrew Gonzalez[1], Jose Angel Regla-Nava[1,†], Erin Maule[1], Michael N Nguyen[1], Norazizah Shafee[1], Sara Landeras Bueno[1], Eduardo Olmedillas[1], Brett Laffey[2], Katarzyna Dobaczewska[2], Zbigniew Mikulski[2], Sara McArdle[2], Sarah R Leist[3], Kenneth Kim[4], Ralph S Baric[3,5], Erica Ollmann Saphire[1,6], Annie Elong Ngono[1*], Sujan Shresta[1*]

[1]Center for Infectious Disease and Vaccine Research, La Jolla Institute for Immunology, La Jolla, CA, USA
[2]Microscopy and Histology Core Facility, La Jolla Institute for Immunology, La Jolla, CA, USA
[3]Department of Epidemiology, University of North Carolina at Chapel Hill, Chapel Hill, NC, USA
[4]Histopathology Core Facility, La Jolla Institute for Immunology, La Jolla, CA, USA
[5]Department of Microbiology and Immunology, University of North Carolina at Chapel Hill, Chapel Hill, NC, USA
[6]Department of Medicine, Division of Infectious Diseases and Global Public Health, University of California, San Diego (UCSD), La Jolla, CA, USA
[†] Current address: Department of Microbiology and Pathology, University Center for Health Science (CUCS), University of Guadalajara, Guadalajara 44340, Mexico
[*]Corresponding authors: Sujan Shresta (sujan@lji.org) and Annie Elong Ngono (aelong@lji.org)

TABLES

| Table S1. Whole-genome sequence homology between SARS-CoV-2 (MT786327) and the 6 other HCoVs[35]. | | | | | | |
|---|---|---|---|---|---|---|
| | **SARS-CoV** (NC_004718) | **MERS-CoV** (NC_019843) | **OC43** (NC_006213) | **HKU1** (NC_006577) | **NL63** (NC_005831) | **229E** (NC_002645) |
| **SARS-CoV-2** | 86.85 | 81.25 | 79.34 | 81.58 | 80.09 | 78.40 |

A

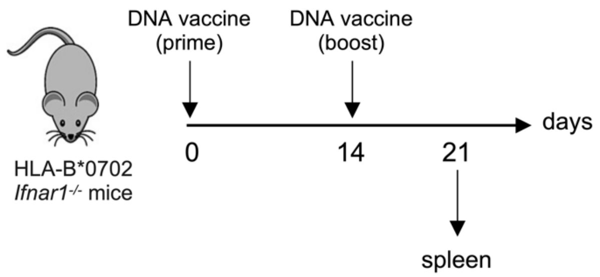


B

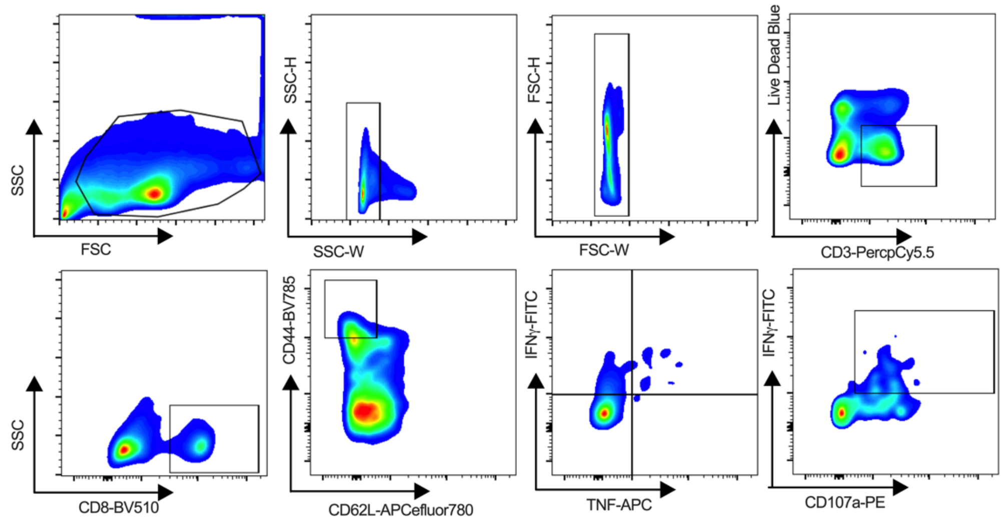


C

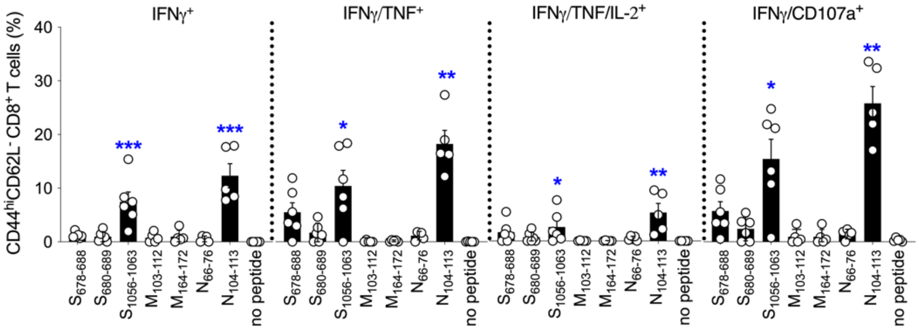


**Figure S1 (related to Figure 1). Validation of SARS-CoV-2 S, N, and M protein-derived epitopes in vaccinated HLA-B*0702 *Ifnar1*$^{-/-}$ mice**

**(A)** Experimental protocol. HLA-B*0702 *Ifnar1*$^{-/-}$ mice were injected with 25 μg S-, N-, or M-based DNA vaccine (*vs* saline) via intramuscular electroporation on days 0 and 14 and spleens collected 7 days later.

**(B)** Gating strategy used to analyze activated ($CD44^+$ $CD62L^-$) $CD8^+$ T cells producing cytokines (IFN$\gamma$, TNF, IL-2) and a degranulation marker (CD107a) after stimulation of splenocytes with SARS-CoV-2-derived peptides. Cells producing $IFN\gamma^+/TNF^+/IL\text{-}2^+$ were identified from the $IFN\gamma^+/TNF^+$ population using a Boolean algorithm.

**(C)** ICS analysis of activated $CD8^+$ T cells in splenocytes stimulated for 6 h with 1 of 7 SARS-CoV-2 S, M, or N protein-derived peptides (*vs* no peptide), immunolabeled for cell surface markers, cytokines, and CD107a, and analyzed by flow cytometry. Data are presented as the mean ± SEM. Circles, individual mice; N=5-6 mice/group pooled from 2 independent experiments. Peptide *vs* control were compared using the Kruskal-Wallis test. *, $P < .05$; **, $P < .01$; ***, $P < .001$.

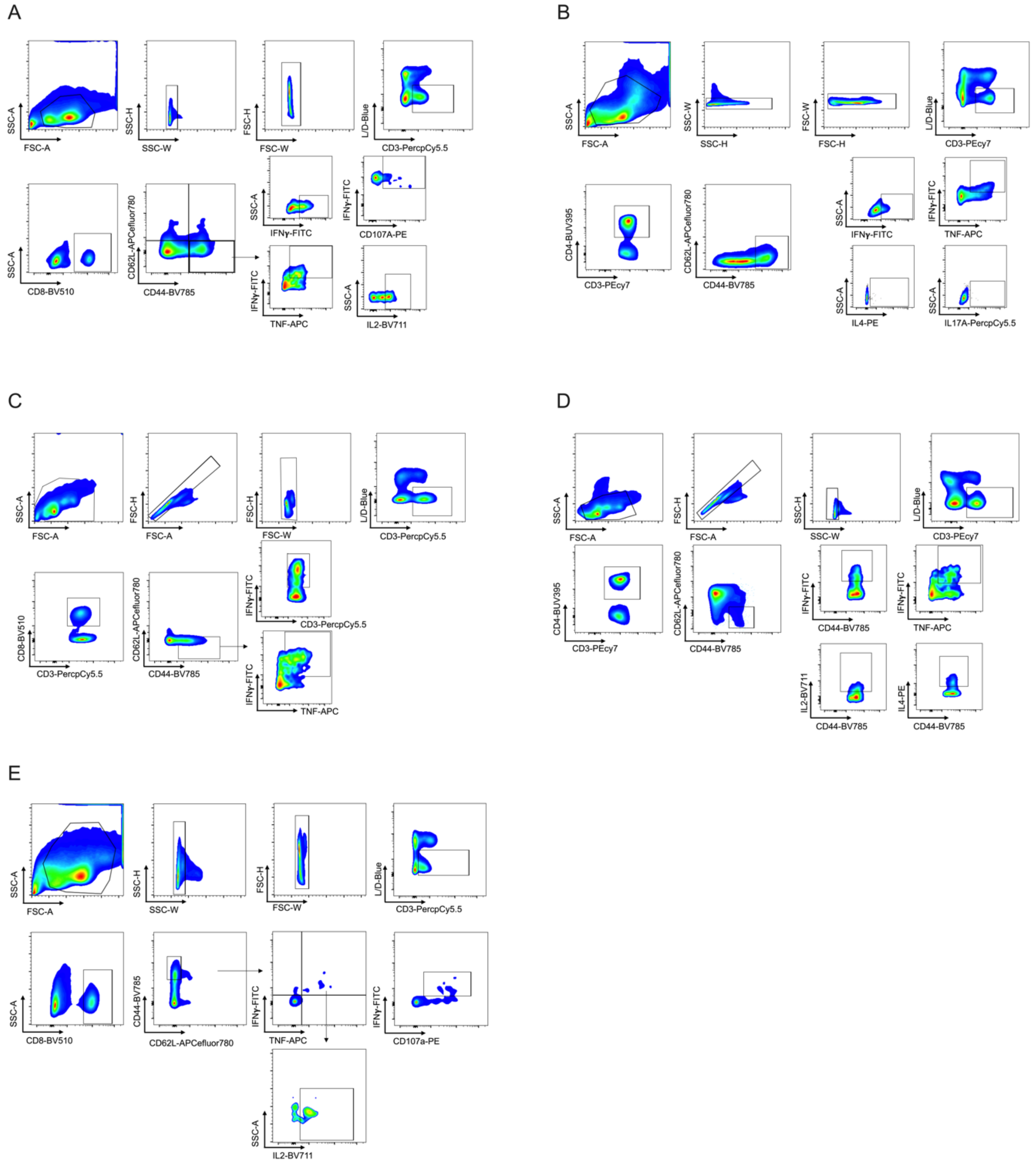


**Figure S2 (related to Figures 2, 3, 4, and 5). Gating strategies used to analyze T cell responses.**

The lymphocyte gate was selected based on size and granularity (FSC and SSC), and double events were excluded using SSC/ FSC height and width. Lymphocytes were selected first for viable $CD3^+$ cells, second for $CD8^+$ or $CD4^+$ subset, and finally for effector memory cells ($CD44^+CD62L^-$). In the effector memory population, polyfunctional T cells were examined for

expression of different cytokines (IFN$\gamma$, TNF, IL-2, IL-4, IL-17A) and degranulation marker (CD107a). For each sample, no less than 50,000 events were acquired within the region of viable $CD3^+$ lymphocytes.

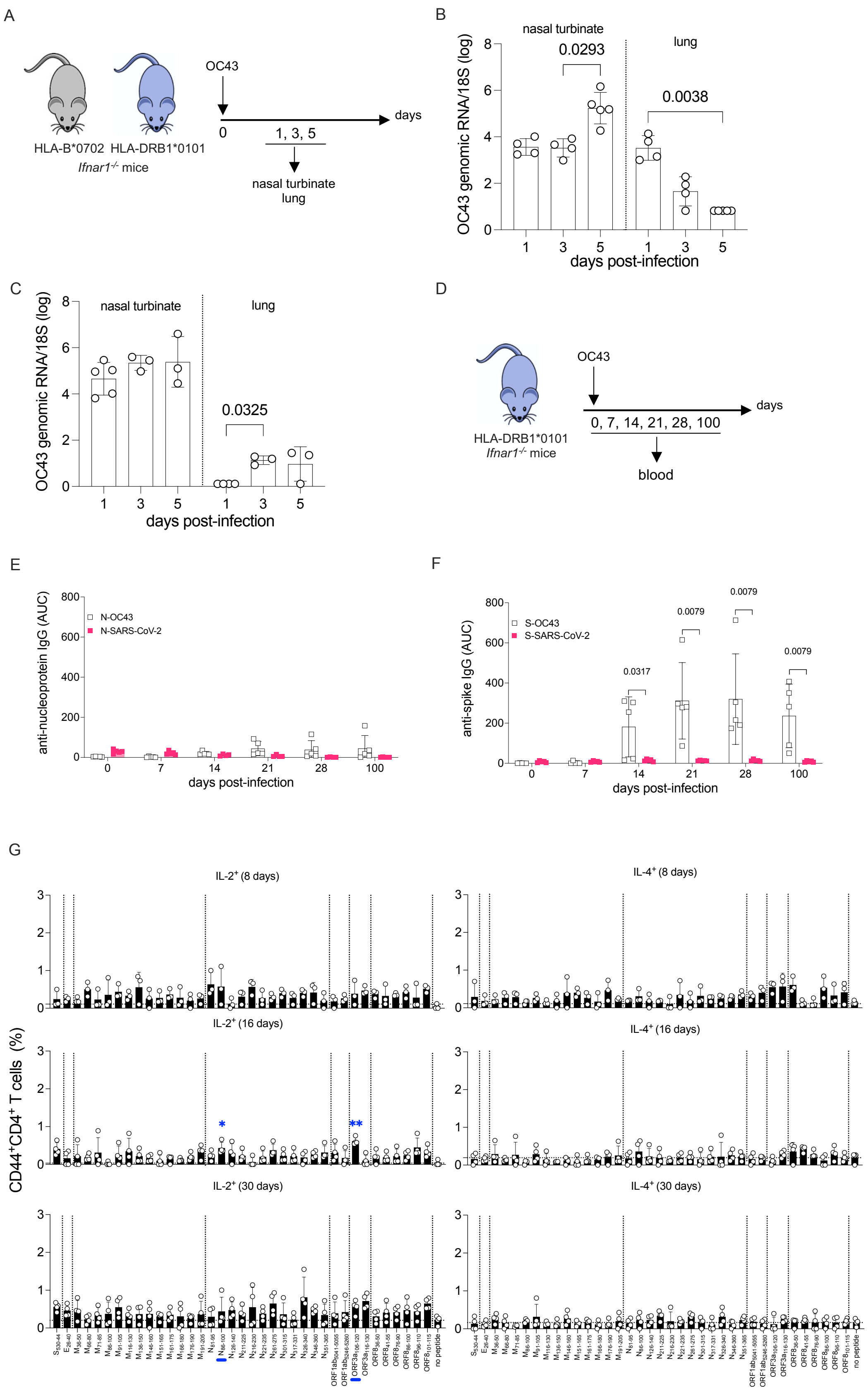
A
HLA-B*0702 HLA-DRB1*0101
Ifnar1-/- mice
OC43
days
1, 3, 5
nasal turbinate
lung
B
nasal turbinate
lung
0.0293
0.0038
OC43 genomic RNA/18S (log)
days post-infection
C
nasal turbinate
lung
0.0325
OC43 genomic RNA/18S (log)
days post-infection
D
OC43
days
0, 7, 14, 21, 28, 100
HLA-DRB1*0101
Ifnar1-/- mice
blood
E
anti-nucleoprotein IgG (AUC)
N-OC43
N-SARS-CoV-2
days post-infection
F
anti-spike IgG (AUC)
S-OC43
S-SARS-CoV-2
0.0317
0.0079
0.0079
0.0079
days post-infection
G
IL-2+ (8 days)
IL-4+ (8 days)
IL-2+ (16 days)
IL-4+ (16 days)
IL-2+ (30 days)
IL-4+ (30 days)
CD44+CD4+ T cells (%)
no peptide

**Figure S3 (related to Figures 3 and 4). OC43 infection in mice.**

**(A)** Experimental protocol for B and C. HLA-B*0702 and HLA-DRB1*0101 *Ifnar1*$^{-/-}$ mice were infected with OC43 ($10^9$ GE, IN) and nasal turbinates and lungs collected on days 1, 3, and 5 post-infection.

**(B and C)** RT-qPCR analysis of genomic OC43 RNA in tissues of HLA-B*0702 *Ifnar1*$^{-/-}$ (B) and HLA-DRB1*01010 *Ifnar1*$^{-/-}$ (C) mice. N=3-5 mice/group. Data were compared using the nonparametric Kruskal-Wallis test.

**(D)** Experimental protocol for E and F. HLA-DRB1*0101 *Ifnar1*$^{-/-}$ mice were infected with OC43 ($10^9$ GE, IN), and blood samples were collected immediately prior to infection, and at 5 time points post-infection.

**(E and F)** ELISA analysis of IgG against S protein (E) and N protein (F) of OC43 and SARS-CoV-2. N=4-5 mice/group. IgG levels against antigens from SARS-CoV-2 *vs* OC43 at each time point were compared using the nonparametric Mann-Whitney test.

**(G)** ICS analysis of activated $CD4^+$ T cells. HLA-DRB1*0101 *Ifnar1*$^{-/-}$ mice were infected with OC43 ($10^9$ GE, IN), and spleens were collected on day 8 (N=3), 16 (N=4), or 30 (N=4) post-infection. Splenocytes were stimulated for 6 h with 37 SARS-CoV-2 peptides (Table 5) *vs* no peptide, immunolabeled for cell surface markers and intracellular cytokines, and analyzed by flow cytometry. Peptide *vs* control data were compared using the nonparametric Kruskal-Wallis test. Blue bars on the x-axis, peptides that significantly stimulated cells at 1 or more time points.

**(B,C,E-G)** Data, pooled from 2 independent experiments, are presented as the mean ± SEM. *, $P < .05$; **, $P < .01$. Circles, individual mice.

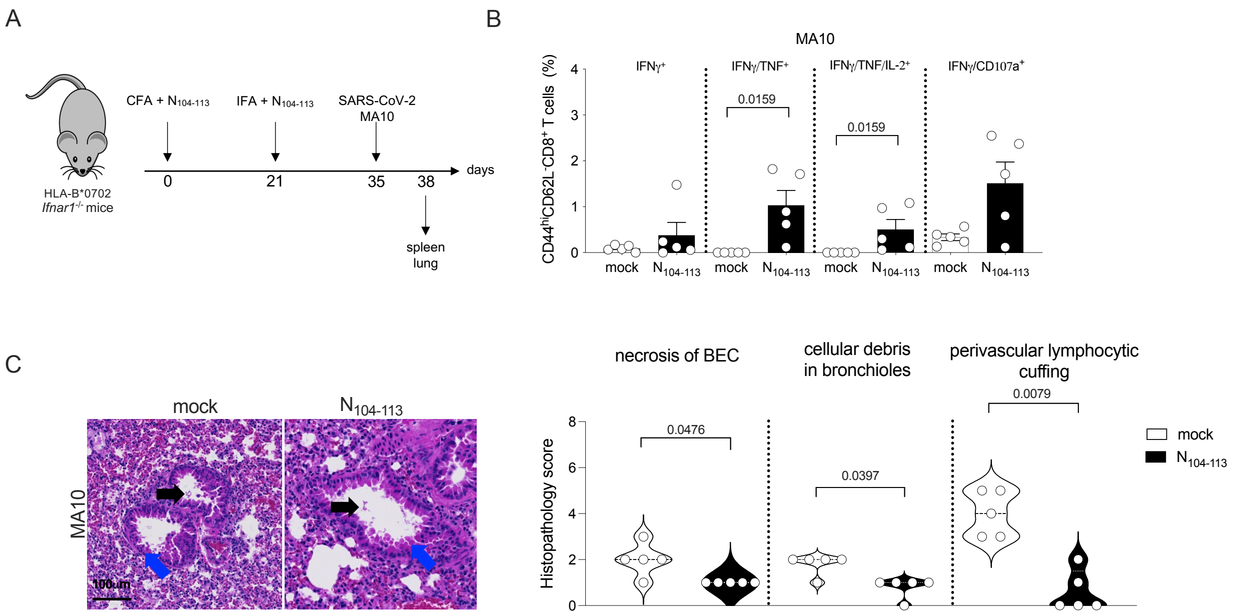


**Figure S4 (related to Figure 5). SARS-CoV-2 $N_{104\text{-}113}$ immunization is protective against SARS-CoV-2-induced lung disease in HLA-B*0702 *Ifnar1*$^{-/-}$ mice challenged with SARS-CoV-2 MA10.**

**(A)** Experimental protocol for B and C. HLA-B*0702 *Ifnar1*$^{-/-}$ mice were injected with SARS-CoV-2 $N_{104\text{-}113}$ *vs* DMSO (mock) on days 0 (complete Freund's adjuvant, CFA) and 21 (incomplete Freund's adjuvant, IFA); 2 weeks later, mice were challenged with SARS-CoV-2 MA10 ($10^5$ PFU, IN) and tissues collected 3 days later.

**(B)** ICS analysis of activated $CD8^+$ T cells. Splenocytes were stimulated for 6 h with SARS-CoV-2 $N_{104\text{-}113}$ peptide, immunolabeled for cell surface markers, intracellular cytokines, and CD107a, and analyzed by flow cytometry.

**(C)** Representative H&E-stained sections of lungs. Blue arrows, bronchiolar epithelial cells (BEC); black arrows, epithelial cells in bronchioles. Sections were scored from 0 (least severe) to 5 (most severe) for standard histopathological features of SARS-CoV-2-induced lung disease.

**(B,C)** Data, pooled from 2 independent experiments, are presented as the mean ± SEM. Circles, individual mice; N=5 mice/group. Groups were compared by the Mann–Whitney test.

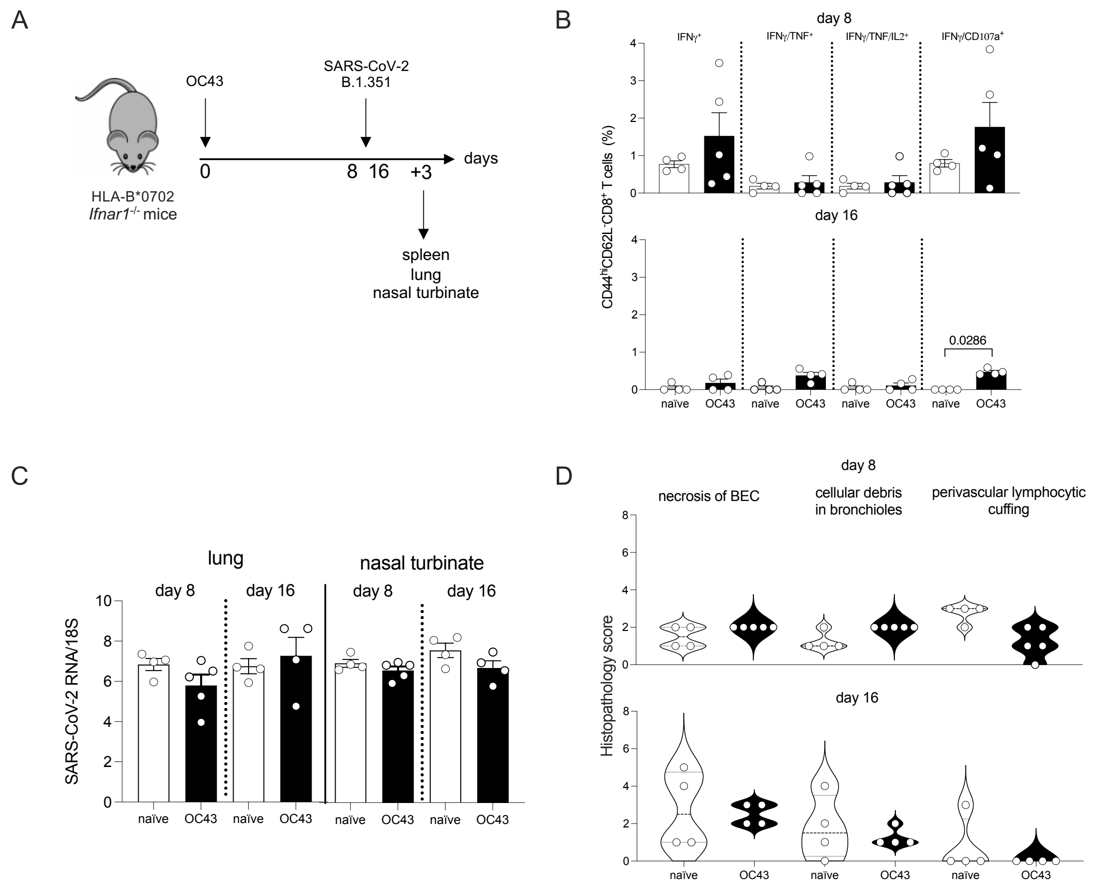


**Figure S5 (related to Figure 5). SARS-CoV-2 RNA load and lung pathology of OC43-infected HLA-B*0702 *Ifnar1*$^{-/-}$ mice challenged with SARS-CoV-2 B.1.351.**

**(A)** Experimental protocol. Mice were infected with OC43 ($10^9$ GE, IN) *vs* medium (naïve), challenged with SARS-CoV-2 B.1.351 ($10^5$ PFU, IN) 8 or 16 days later, and tissues were harvested at 3 days post-challenge.

**(B)** RT-qPCR of genomic OC43 RNA.

**(C)** H&E-stained lung sections were scored from 0 (least severe) to 5 (most severe) for standard histopathological features of SARS-CoV-2-induced lung disease.

**(D)** ICS analysis of OC43-elicited activated CD8$^+$ T cells. Splenocytes were stimulated for 6 h with SARS-CoV-2 $N_{104-113}$ peptide, immunolabeled for cell surface markers, intracellular cytokines, and CD107a, and analyzed by flow cytometry.

**(B-D)** Data, pooled from 2 independent experiments, are presented as the mean ± SEM. Circles, individual mice; n=4-5 mice/group. Groups were compared using the Mann–Whitney test.

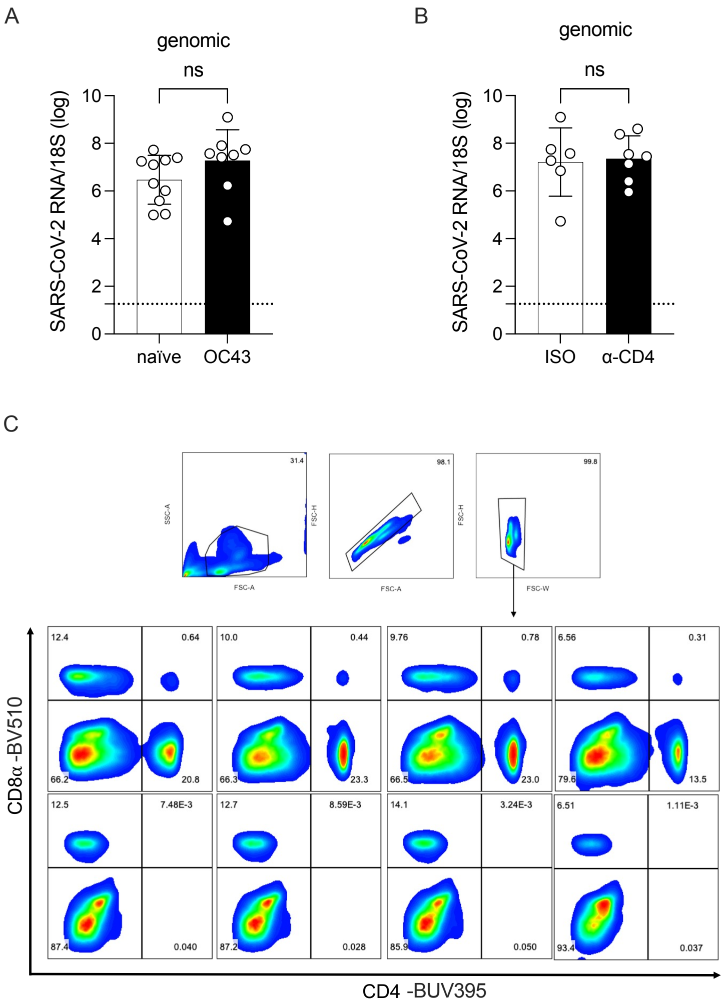


**Figure S6 (related to Figure 6). SARS-CoV-2 challenge of HLA-DRB1*0101 *Ifnar1*$^{-/-}$ mice pre-exposed to OC43.**

Experimental protocols are described in Figures 6A (A) and 6E (B,C).

**(A,B)** RT-qPCR of SARS-CoV-2 genomic RNA in nasal turbinates. Data are presented as the mean ± SEM. Circles, individual mice. Mice/group: A, 8 (OC43-infected) and 10 (naïve); B, 6-7. Group differences were analyzed by the Mann–Whitney test. Horizontal dotted line, the limit of detection.

**(C)** Gating strategy used to analyze CD4$^{+}$ T cells in the blood of mice treated with a CD4$^{+}$ T cell-depleting antibody (α-CD4) vs isotype control. Each dot plot represents a mouse.
